# Multifaceted Defense against *Listeria* *monocytogenes* in the Gastro-Intestinal Lumen

**DOI:** 10.3390/pathogens7010001

**Published:** 2017-12-22

**Authors:** Simone Becattini, Eric G. Pamer

**Affiliations:** 1 Immunology Program, Sloan Kettering Institute, Memorial Sloan-Kettering Cancer Center, New York, NY 10065, USA; 2 Lucille Castori Center for Microbes Inflammation and Cancer, Molecular Microbiology Core Facility, Memorial Sloan-Kettering Cancer Center, New York, NY 10065, USA; 3 Infectious Diseases Service, Department of Medicine, Memorial Sloan-Kettering Cancer Center, New York, NY 10065, USA

**Keywords:** *Listeria monocytogenes*, microbiota, colonization resistance

## Abstract

*Listeria monocytogenes* is a foodborne pathogen that can cause febrile gastroenteritis in healthy subjects and systemic infections in immunocompromised individuals. Despite the high prevalence of *L. monocytogenes* in the environment and frequent contamination of uncooked meat and poultry products, infections with this pathogen are relatively uncommon, suggesting that protective defenses in the general population are effective. In the mammalian gastrointestinal tract, a variety of defense mechanisms prevent *L. monocytogenes* growth, epithelial penetration and systemic dissemination. Among these defenses, colonization resistance mediated by the gut microbiota is crucial in protection against a range of intestinal pathogens, including *L. monocytogenes*. Here we review defined mechanisms of defense against *L. monocytogenes* in the lumen of the gastro-intestinal tract, with particular emphasis on protection conferred by the autochthonous microbiota. We suggest that selected probiotic species derived from the microbiota may be developed for eventual clinical use to enhance resistance against *L. monocytogenes* infections.

## 1. Introduction

*Listeria monocytogenes* is a gram-positive pathogen that infects humans and animals by ingestion of contaminated food, and is associated with high rates of mortality [1,2]. While *Listeria* outbreaks are common and generally attributable to highly contaminated foods, most cases of listeriosis are sporadic [3].

From the intestinal lumen, *L. monocytogenes* can cross the epithelial layer via a variety of mechanisms, including receptor-dependent entry into Goblet and epithelial cells, as well as trancytosis through M cells and penetration into underlying tissues [4,5,6,7,8,9,10,11]. Systemic spread of *L. monocytogenes* can have particularly severe consequences for selected cohorts of individuals, such as immunocompromised and cancer patients, pregnant women, young children and the elderly [1,2]. If ingested at high doses, *L. monocytogenes* can also induce febrile gastro-enteritis in healthy individuals [12,13]. High pathogen load in food is generally the result of contamination that occurs in food-manufacturing plants (cheese, packaged meat, pre-washed and pre-sliced processed produce), which is mainly due to *L. monocytogenes* dispersion in livestock and the environment. Several studies have identified *L. monocytogenes* in feces of a high percentage of dairy and beef cattle and in sheep [14,15,16]. Furthermore, *L. monocytogenes* is commonly found in sewage sludge [17], which is occasionally used to fertilize agricultural land. Thus, *L. monocytogenes* is widely distributed in the environment and frequently contaminates foods, making encounter with this pathogen a relatively common event for humans.

Although it is not widely appreciated, *Listeria monocytogenes* transiently and asymptomatically colonizes a sizable percentage of the human population, with estimates varying between 1 and 5% across studies [15,18,19,20,21,22,23]. The frequency of colonization with *L. monocytogenes* exceeds the reported incidence of Listeriosis by orders of magnitude [2,3]. This discrepancy might be the result of undiagnosed cases of Listeriosis, as symptoms of the infection can be mild and resolve spontaneously, making blood culturing and hospital admission unnecessary, particularly with respect to the *L. monocytogenes*-induced enteritis. Furthermore, ad hoc detection tests for *L. monocytogenes* are not routinely performed in hospitalized subjects, as suggested elsewhere [24,25]. However, a more likely possibility is that despite frequent passages through the GI tract of human beings, *L. monocytogenes* rarely progresses to systemic infection. This would suggest that efficient resistance mechanisms are in place to prevent expansion of this pathogen following ingestion. Among other factors, the presence of a healthy gut microbiota, which can provide colonization resistance against a variety of enteric pathogens, may be particularly effective. Here we review evidence from studies conducted over the past five decades that support a key role for host-derived as well as commensal microbe-associated factors in providing protection against intestinal colonization and systemic infection with *Listeria monocytogenes*.

## 2. A Brief Overview of In Vivo Colonization by *L. monocytogenes*

The mechanisms of *Listeria* invasion and spread within the mammalian host have been elucidated by elegant experimental studies mapping the trafficking of genetically tagged bacteria upon inoculation into mice and guinea pigs [26,27]. These studies have demonstrated that *L. monocytogenes* replicates in intestinal villi and is shed into the gut lumen, generating a second wave of penetration that results in Peyer’s Patch invasion. Subsequently, *L. monocytogenes* accesses mesenteric lymph nodes and disseminates to spleen and liver, also colonizing the gallbladder. *L. monocytogenes* replicates in the gallbladder and re-accesses the intestinal lumen via the common bile duct upon gallbladder contraction [26,27].

Although most studies have focused on *L. monocytogenes* colonization and invasion of the small intestine, several studies, including our own, have demonstrated that marked expansion and prolonged persistence of *L. monocytogenes* occurs in the large intestine of rodents as well as other model animals [10,28,29,30]. Thus, the large intestine, rather than the small intestine, may be the major portal of dissemination for *L. monocytogenes*, consistent with the high local density of Goblet cells, which have been shown to be permissive for pathogen trans-cytosis [31]. Intra-rectal administration of *Listeria* results in systemic infection of mice following dissemination via caudal lymph nodes [29,32], supporting the notion that the large intestine serves as a likely invasion portal.

## 3. Defense against *Listeria monocytogenes* in the Gastro-Intestinal Lumen

### 3.1. Host-Derived Factors

#### 3.1.1. Host Physical and Chemical (Non-Immune) Defenses

Mice and humans are overall highly resistant to *L. monocytogenes* infection via the oral route [33]. This is likely due to a variety of factors, including the presence of multiple lines of defense along the host’s GI tract. To begin with, there are a number of physical and chemical defenses that the gastrointestinal milieu engages against foodborne pathogens, and in response to which *L. monocytogenes* expresses an array of molecular weapons, extensively reviewed elsewhere [34]. Activation of dedicated stress responses in *Listeria*, which allow bacterial survival in the intestinal lumen, results from the enlistment of the alternative RNA polymerase factor SigB and on the virulence regulator prfA, both of which are induced upon passage through the mammalian GI tract [35,36].

Briefly, upon ingestion and transit to the stomach and duodenum, *L. monocytogenes* is first exposed to low pH gastric acids, whose bactericidal activity is potent with respect to this particular pathogen [36]. Accordingly, both humans and animals appear to have increased susceptibility to infection upon treatment with drugs that reduce gastric acidity [37,38]. As a result of gastric acid-mediated killing of *L. monocytogenes*, only a fraction of the infectious inoculum enters the small intestine of laboratory animals in the minutes to hours following the infection [39], providing at least a partial explanation for the need to use high doses of *L. monocytogenes* to induce disease in healthy individuals. Importantly, *L. monocytogenes* undergoes a “stress hardening” response in that exposure to moderately low pH (~5.5) increases its resistance to otherwise lethal pH (~3.5) [34]. This process was shown to increase viability of the pathogen in vivo after oral inoculation, including favoring spread of live bacteria to the mesenteric lymph nodes [40]. Resistance against low pH in the stomach and duodenum is achieved by *L. monocytogenes* through several mechanisms, including import and decarboxylation of glutamate and catabolism of arginine to ornithine, both resulting in consumption of protons and increases in intracellular pH [41,42,43].

Upon passage to the duodenum, *Listeria* also utilizes several transporters to resist osmotic stress, as salt concentration in the intestine is relatively high [34]. Bile salts also represent a defensive antibacterial mechanism that protects the host by preventing expansion of certain pathogens [44,45,46]. *Listeria*, however, can grow directly in the gallbladder of humans and mice, in the presence of high concentrations of bile salts [47], and re-enter the intestinal lumen via the bile duct [48]. The high levels of resistance to bile are, in part, conferred by expression of bile-salts hydrolases, which are up-regulated in hypoxic conditions [49,50,51], as well as bile exclusion systems [52,53].

To persist in the gut, *L. monocytogenes* must at least partially resist expulsion by intestinal peristalsis. Adhesion to the intestinal epithelium enables *L. monocytogenes* to delay expulsion and is regulated by the virulence factor ActA [54] and the adhesion protein LAP [55], among others. In order to disseminate from the gut, *L. monocytogenes* must overcome the physical barriers represented by the mucus layer and the intestinal epithelium, respectively [56]. Several proteins encoded by the genome of *Listeria* isolates, including internalins, have been shown to bind host mucins, and might therefore be involved in adherence to the mucus layer [57,58,59]. Listeriolysin O (LLO), the major virulence factor secreted by *L. monocytogenes* that enables it to access the cytoplasm of infected cells, can compromise barrier functions of epithelial monolayers [60]. Interestingly, epithelial cells respond to exposure to LLO by increasing mucin production, thereby reinforcing the barrier against *L. monocytogenes* invasion [61,62]. Intestinal P glycoprotein also provides a defense against *L. monocytogenes* infection, through mechanisms that remain to be elucidated, and mice lacking this protein show higher bacterial dissemination following oral challenge [63].

#### 3.1.2. Host Immune Defenses

Multiple immune mechanisms also contribute to reducing *L. monocytogenes* density in the gastro-intestinal lumen. Although mucosal antibodies are produced in response to *L. monocytogenes* infection [64], these are mostly not protective, and intestinal clearance of *Listeria* occurs with similar kinetics in WT and Rag-deficient mice, thus ruling out a prominent role for immunoglobulins in pathogen expulsion [28]. In contrast, innate immune mechanisms have been consistently reported to effectively reduce *L. monocytogenes* expansion in the gut lumen. One of the most studied among such mechanisms is production of anti-bacterial peptides by Paneth and epithelial cells. RegIIIγ is a bactericidal lectin whose production is induced by microbial stimuli, via signaling through the adaptor protein MyD88 and production of IL-23 and IL-22 [39,65]. Using a mouse model utilizing ileal loop ligation, RegIIIγ was shown to kill *L. monocytogenes* in the small intestinal lumen [39]. More recent studies have demonstrated that proteins of the RegIII family form a pore in the bacterial cell membrane [66]. Crypdtidins (called α-defensins in humans) are anti-microbial peptides produced in the intestine, in a partially NOD2 dependent manner. Although absence of NOD2 signaling reduces expression of some cryptidins and increases susceptibility to systemic *L. monocytogenes* infection following intra-gastric inoculation, it is unclear whether this involves enhanced bacterial growth in the intestinal lumen [67]. Along similar lines, a porcine β-defensin homologous to human β-defensin 1 was found to have anti-*Listerial* activity in vitro [68], and similar results were obtained with intestinal phospholipase A2 (iPLA2) [69].

The antimicrobial peptide CRAMP, which protects the intestinal compartment during early life prior to the development of Paneth cells, increases resistance to orally inoculated *Listeria* [70]. Of note, Paneth cell-secreted anti-microbial peptides are only effective in close proximity to the crypt or within the mucus layer, but not in the intestinal lumen [71]. It is likely, therefore, that other mechanisms prevent uncontrolled growth of *L. monocytogenes* in the intestinal lumen.

### 3.2. Gut Microbiota

#### 3.2.1. Evidences for a Role of Gut Microbiota in Protection against *Listeria monocytogenes*

The intestinal microbiota can directly and indirectly influence most of the protective immune mechanisms mentioned above, by providing tonic signals to the host’s mucosal epithelial cells and the innate and adaptive immune systems. For instance, production of mucus [72] and anti-microbial peptides [73], modification of conjugated primary bile salts [74], hematopoiesis [75] and functional maturation of immune cells [76], are all heavily influenced by gut commensal bacteria (reviewed in [77]). The protective role of commensal bacteria has been demonstrated in the setting of gastrointestinal *L. monocytogenes* infection. For example, commensal bacteria are necessary for correct cargo sorting in Paneth cells, and depletion of microbiota through antibiotic treatment results in lysosomal degradation of lysozyme, which increases both intestinal and systemic invasion of *Listeria monocytogenes* [78]. The microbiota also induces up-regulation of the surface molecule SLAM4 selectively on intestinal lymphocytes, and this promotes resistance to oral challenge with *Listeria monocytogenes* [79]. Some *Lactobacilli* have been shown to modulate host immune responses in the intestine, leading to a small but significant reduction in the expansion and dissemination of orally inoculated *Listeria* [80]. Similarly it was shown that, in vitro, *Lactobacilli* and *Bifidobacteria* can prevent *Listeria* invasion of epithelial cells, both by secreting anti-bacterial factors, as well as by modulating epithelial cytokine production [81].

Aside from these indirect effects on host metabolism and immune development, the microbiota provides direct colonization resistance against exogenously acquired bacteria. Indeed, colonization resistance represents arguably the most important benefit that the host derives from its symbiosis with commensal bacteria [82]. Classical experiments from more than 60 years ago demonstrated that antibiotic-mediated depletion of the microbiota markedly enhances susceptibility to bacterial (*Salmonella*) infections [83]. However, only in recent years have the underlying mechanisms been elucidated. It is now appreciated that different bacterial species provide resistance to specific pathogens through distinct mechanisms. Thus, antibiotics with distinct antibacterial spectra can differ in terms of inducing susceptibility to colonization and infection with different pathogens [44,84].

*L. monocytogenes* is also vulnerable to microbiota-mediated colonization resistance and commensal microbes can be highly effective at reducing its expansion in the intestinal lumen. The first demonstration of this concept was provided by experiments comparing the efficiency and severity of oral *L. monocytogenes* infection in germ-free (GF) versus specific-pathogen free (SPF) rodents [85,86]. GF mice and rats, in contrast to animals bearing an intact autochthonous microbiota, were highly susceptible to extremely low doses of *Listeria*, [85,86]. Although suboptimal immune development in GF animals [87] might have contributed to these results, it is now appreciated that immune mechanisms are unlikely to completely mediate the dramatic inhibition of *L. monocytogenes* expansion in the gut lumen of SPF compared to GF animals (8–10 orders of magnitude) [39,85,86]. The early studies with GF animals correctly hypothesized that the microbiota plays a major, direct role in providing colonization resistance against *L. monocytogenes*.

Initial attempts to protect GF mice by transferring commensal Clostridium and a Bacteroides species prior to *L. monocytogenes* infection were only partially successful and demonstrated that the pathogen still expanded to high densities in the lumen, but that the number of bacteria near the intestinal mucus layer was reduced in the presence of these commensals [85]. Given the remarkable diversity of the “healthy” intestinal microbiota, the incomplete effectiveness of the two selected commensal bacterial strains likely reflects the requirement for additional bacterial species to provide complete protection. Indeed, swabbing the mouth of *L. monocytogenes*-monoassociated gnotobiotic rats with a suspension of feces from conventional SPF animals was sufficient to reduce *Listeria* luminal density by about seven orders of magnitude within a day, and to obtain complete pathogen clearance in a few weeks [86]. Further supporting a role for the microbiota in providing protection against intestinal *L. monocytogenes* infection, it was noted that prolonged starvation, which is known to perturb the composition of intestinal commensal communities, predisposes guinea pigs to listeriosis [85]. Along similar lines, a study in rats showed that a higher percentage of animals become infected upon pre-treatment with antibiotics [88]. Furthermore, treatment with cephalosporins (a class of eta-lactam antibiotics to which *L. monocytogenes* is resistant) was shown to worsen the effect of immune-suppressant drugs administered prior to oral *L. monocytogenes* inoculation, by increasing both intestinal expansion and systemic spread of intragastrically inoculated bacteria [89].

Consistent with this previous work, we recently showed that perturbation of the microbiota through antibiotic treatment dramatically increases luminal expansion of *L. monocytogenes* in mice, rendering doses as low as 100 CFUs capable of colonizing the entire intestinal tract and leading to systemic spread [28]. Disruption of microbiota-mediated colonization resistance dramatically increased susceptibility of immunocompromised hosts, such as Rag/common γ chain-deficient mice, which lack B, T and natural killer (NK) cells and innate lymphocytes (ILCs) to *L. monocytogenes* infection. Mice treated with anti-cancer chemotherapy and rendered leukopenic maintained resistance to low-dose oral *L. monocytogenes* infection unless the microbiota was disrupted with antibiotics, in which case there was a marked increase in bacterial dissemination from the gut leading to increased mortality. These immunocompromised mice succumbed to infection with *Listeria* inocula as low as 10^4^ CFUs, indicating that the in the absence of a functional immune system, the microbiota plays a non-redundant and critical protective role [28]. These findings are of clinical relevance, as immunocompromised patients can develop systemic *L. monocytogenes* infections that are sporadic, i.e., not associated with large outbreaks, and thus likely result from the ingestion of foods that are contaminated with relatively low densities of *L. monocytogenes*.

The intestinal content of mice efficiently suppresses growth of *Listeria monocytogenes* in anaerobic co-culture systems. This inhibition is complex in that supernatants of cultures of small intestinal content inhibit *L. monocytogenes* growth, while inhibition by colonic bacteria requires bacterial co-cultures and thus is not mediated by a secreted product [28]. This difference likely reflects the higher density of *Lactobacilli* in the small intestine, which produce bacteriocins capable of killing *Listeria monocytogenes* [90,91,92,93]. We recently identified bacterial taxa that provide colonization resistance against *L. monocytogenes*, and assembled a consortium of four *Clostridiales* species that efficiently protect ex-GF mice, upon reconstitution, from orally administered *L. monocytogenes*. In contrast, GF mice reconstituted with dysbiotic microbial consortia recovered from antibiotic-treated mice had marked luminal replication of *L. monocytogenes* with dissemination to the spleen and liver [28]. Since isolated bacterial strains and commensals from antibiotic-treated mice have also been shown to promote *L. monocytogenes* expansion [28,94], it is clear that the gut microbiota, depending on its composition, can increase resistance or enhance susceptibility to infection. Because dysbiosis can dramatically impact susceptibility to *L. monocytogenes* infection, we propose that in individuals at high risk for infection, gut microbiota composition might represent an important predisposing factor. Characteristics such as advanced age [95,96], altered physiological status (for instance pregnancy) [97] and use of immunosuppressive medications [98] are known to alter the structure of commensal communities and are associated with increased susceptibility to listeriosis, raising the possibility that changes in the microbiota represent an underlying cause of this association.

Recent work from a number of laboratories strongly suggests that commensal bacteria might be developed as probiotics that could facilitate the prevention or even treatment of gastrointestinal Listeriosis. Interestingly, colonization resistance against *L. monocytogenes* has likely been serendipitously exploited in the poultry industry, by the provision of commercially available competitive exclusion (CE) cultures, i.e., preparations of commensal bacteria from cecal contents of adult healthy broiler chickens [99]. Such products have been used successfully to prevent expansion of human pathogens (particularly *Salmonella* species) in the intestine of chickens in the poultry industry, with protection being provided by a handful of bacterial strains later identified through in vitro screenings [100]. As for *L. monocytogenes*, one study showed that when gavaged into young chickens prior to challenge, CE cultures prevented intestinal expansion of the pathogen in 100% of the cases [101].

#### 3.2.2. Possible Mechanisms Involved in Microbiota-Mediated Colonization Resistance against *Listeria monocytogenes*

Direct microbiota-mediated inhibition of *Listeria monocytogenes* can be potentially achieved through different mechanisms, of which the best studied is production of anti-microbial molecules. These include bacteriocins, mainly produced by the genera lactobacillus, lactococcus, enterococcus and bifidobacterium [102].

Many bacteriocins have been shown to be highly active against *L. monocytogenes* in vitro [90,91,92,93]. Importantly, multiple bacterial strains producing anti-listerial bacteriocins could be isolated from cultures of human stool, leading to the suggestion that those strains might be useful as probiotics to prevent or treat Listeriosis [103,104,105,106].

Lantibiotics are a class of bacteriocins/antibiotics that seem to be particularly effective against *Listeria monocytogenes*. Nisins, the prototypical lantibiotics, are produced by *L. lactis* and other commensals, and have been approved by the FDA (US Food and Drug Administration) as a food preservative [107]. The isotype nisin V, in particular, has pronounced anti-*Listerial* activity both *in vitro* and *in vivo* [108,109,110]. Formicin, another recently identified lantibiotic, also inhibits *L. monocytogenes* growth [111].

Although bacteriocin production correlates with *in vitro Listeria* inhibition, *in vivo* experiments demonstrating the efficacy of endogenous production of these molecules (i.e., intestinal reconstitution of model animals with protective strains) are for the most part lacking in the literature. One notable exception is the work of Gahan and collaborators, who showed that daily gavage with a *Lactobacillus salivarius* strain producing the Abp118 bacteriocin conferred resistance to *Listeria* infection in mice [112].

Bacterial soluble mediators other than bacteriocins have also been reported to be active against *Listeria*. *Escherichia coli* Nissle 1917, a probiotic strain that has been tested in clinical trials, prevents *L. monocytogenes* entry into intestinal cell lines, without affecting pathogen’s viability, through a contact-independent mechanism [113].

Alternative soluble mediators of microbial origin that can protect against *L. monocytogenes* include short chain fatty acids [114]. *Veillonellae* were shown to inhibit *L. monocytogenes* in the presence of high concentrations of tartrate, which enhanced the production of propionate and acetate, thus decreasing the pH of the culture [115]. This suggests that diet could also affect the capacity of specific commensals to promote colonization resistance against *L. monocytogenes*.

Mechanisms of bacterial suppression of *L. monocytogenes* that do not rely on the release of soluble mediators might include nutrient competition and contact-dependent mechanisms. Contact dependent inhibition (CDI) mechanisms are known to operate among Gram negative bacterial species [116,117]. Although the existence of CDI in Gram+ bacteria, and in particular against *Listeria monocytogenes*, had been postulated based on experimental results [28,118,119], no mechanism had been proposed until recently. Pioneering work from Mougous and collaborators has now definitively proven CDI to exist also among Gram positive bacteria, and has begun to reveal the molecular mechanisms [120]. Briefly, toxins of the LXG protein family can be produced by Gram positive bacteria and are translocated into neighboring Gram positive cells through the Esx transport system, where they promote degradation of lipid II and consequently inhibit peptidoglycan biosynthesis. Specific immunity genes provide resistance to these toxins in the producing strains. Importantly, this study identified LXG toxins as widely distributed across Firmicutes in the human intestinal microbiota, suggesting a potential role for these molecules not only in shaping the composition of the bacterial community, but possibly also in providing colonization resistance against Gram positive pathogens [120].

Of note, bacteria belonging to the *Listeria* genus encode CDI-mediating LXG toxins [121]. Furthermore, epidemic *L. monocytogenes* strains have been shown to carry Listeriolin S (LLS), which favors luminal expansion of the pathogen and tissue invasion, likely via elimination of competing commensals [122]. Thus, it appears that *L. monocytogenes* is endowed with a range of defenses to combat commensals and promote its own survival in the intestine.

## 4. Conclusions

Luminal expansion of *L. monocytogenes* in the gut increases the risk of systemic dissemination and the development of severe disease, and classic and more recent experimental studies using animal models are revealing that host defense is multifaceted, involving the epithelial barrier, innate and adaptive immune defenses and, perhaps least appreciated but extremely important, the commensal microbial populations that inhabit the mammalian GI tract. Protective mechanisms include the low pH and high osmolarity of gastric juices and intestinal content, bile acids, antimicrobial molecules of host origin and those produced by the autochtonous microbiota. The healthy gut microbiota utilizes multiple strategies to effectively reducing *Listeria monocytogenes* survival upon oral ingestion. We propose that microbiota-mediated colonization resistance represents the main mechanism by which hosts are rendered highly resistant to frequent encounters with *L. monocytogenes*. If this hypothesis were correct, two corollaries would be particularly relevant: first, subjects considered at risk should be spared as much as possible from microbiota-perturbing interventions; second, provision of specific probiotics might benefit highly susceptible individuals as well as the general population, especially during outbreaks of *L. monocytogenes* infection. Because intestinal commensal bacterial species are non-pathogenic and readily culturable, their use to prevent and possibly treat early phases of *Listeria monocytogenes* infection may have significant clinical utility. Of note, expanding on our work [28], it can be envisioned that a probiotic consortium composed by multiple commensal bacteria capable of inhibiting *L. monocytogenes* via distinct and synergistic mechanisms might provide high-level resistance to this pathogen.
